# Infiltrating T cells promote renal cell carcinoma (RCC) progression *via* altering the estrogen receptor β-DAB2IP signals

**DOI:** 10.18632/oncotarget.5884

**Published:** 2015-11-17

**Authors:** Chiuan-Ren Yeh, Zheng-Yu Ou, Guang-Qian Xiao, Elizabeth Guancial, Shuyuan Yeh

**Affiliations:** ^1^ Department of Urology, University of Rochester Medical Center, Rochester, NY 14642, USA; ^2^ Department of Pathology, University of Rochester Medical Center, Rochester, NY 14642, USA; ^3^ Department of Medicine, University of Rochester Medical Center, Rochester, NY 14642, USA

**Keywords:** ERβ, RCC, metastasis, DAB2IP, CD4+ T cells

## Abstract

Previous studies indicated the T cells, one of the most common types of immune cells existing in the microenvironment of renal cell carcinoma (RCC), may influence the progression of RCC. The potential linkage of T cells and the estrogen receptor beta (ERβ), a key player to impact RCC progression, however, remains unclear. Our results demonstrate that RCC cells can recruit more T cells than non-malignant kidney cells. Using an *in vitro* matrigel invasion system, we found infiltrating T cells could promote RCC cells invasion *via* increasing ERβ expression and transcriptional activity. Mechanism dissection suggested that co-culturing T cells with RCC cells released more T cell attraction factors, including IFN-γ, CCL3 and CCL5, suggesting a positive regulatory feed-back mechanism. Meanwhile, infiltrating T cells may also promote RCC cell invasion *via* increased ERβ and decreased DAB2IP expressions, and knocking down DAB2IP can then reverse the T cells-promoted RCC cell invasion. Together, our results suggest that infiltrating T cells may promote RCC cell invasion *via* increasing the RCC cell ERβ expression to inhibit the tumor suppressor DAB2IP signals. Further mechanism dissection showed that co-culturing T cells with RCC cells could produce more IGF-1 and FGF-7, which may enhance the ERβ transcriptional activity. The newly identified relationship between infiltrating T cells/ERβ/DAB2IP signals may provide a novel therapeutic target in the development of agents against RCC.

## INTRODUCTION

Renal cell carcinoma (RCC) is the most common kidney cancer and third leading cause of death among urological tumors [1]. There are four subtypes of RCC and clear cell tumor subtype is the most common type, accounting for 80% of all RCCs [2]. The inactivating mutation in the von Hippel–Lindau (VHL) gene, a tumor suppressor, is frequently observed in clear cell RCC [3]. RCC patients can easily develop resistance to chemotherapy and radiation therapy, therefore kidney resection is considered to be the effective treatment for patients with clinically localized RCC. Unfortunately, the postoperative recurrence in RCC patients who undergo curative nephrectomy is only around 20% to 40% and rarely curable [4]. Thus, there is a clear need to improve treatment options for metastatic RCC.

The tumor microenvironment (TME) with infiltrated immune cells may play key roles in tumor progression [5]. RCC has been well recognized as an immune escape disease. This could be due to alterations in the tumor itself resulting in its impaired recognition by the immune system or by the dysfunction of immune cells in RCC patients [6]. Furthermore, immune dysfunction contributing to tumor invasion has been reported in some RCC patients [7]. The most common types of immune cells in RCC tumors are T cells and natural killer cells [8]. Regulatory T cells (Treg), a subpopulation of CD4+ T cells, have been demonstrated to suppress the self-reactive T cells [9, 10]. Furthermore, it is reported that Treg cells have a modest enrichment among these tumor recruited T lymphocytes [11]. Importantly, the increased Treg cells population is found to be associated with a poor prognosis in women with advanced ovarian cancer and was implicated in preventing the induction of effective antitumor immunity [12]. In the present report, we study the novel mechanisms by which how T cells regulate RCC invasion.

The effects of estrogens are evident through their binding to estrogen receptors (ERs) and subsequent regulation of the transcription and activation of downstream genes. There are two subtypes of ERs, ER alpha and beta (ERα and ERβ). The distribution of ERα and ERβ varies in different tissue types and affect tumor progression, including breast, prostate and bladder cancers [13–19]. It was reported that there is only ERβ, but no ERα, expression in cultures of RCC cells and in human RCC tissues [20]. The ERβ roles in RCC progression, however, remain to be further studied. In this study, we investigate the role of recruited T cells in RCC and their potential linkage to ERβ expression in promoting the RCC cell invasion.

## RESULTS

### RCC cells can better attract CD4+ T cells than the non-malignant kidney cells

**Figure 1:**
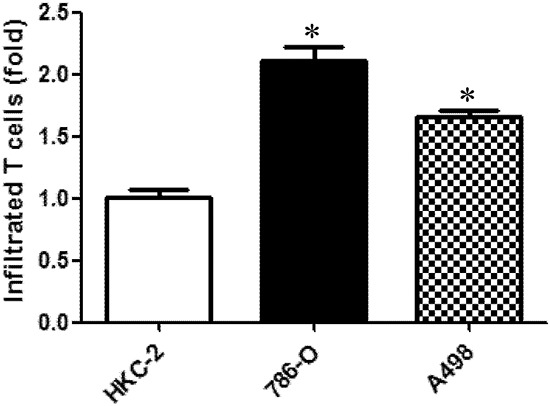
RCC cells can better attract CD4+ T cells than the non-malignant kidney cells Transwell migration assay was applied to study the capability of RCC cell to attract T cells. Non-malignant kidney cells (HKC2) and RCC cells (786-O and A498) were seeded in the lower chamber of transwells, and T cells were placed in the upper chambers with 5 μm pore size members. After 6 hrs of incubation, the media in the bottom well was collected and numbers of migrated T cells were counted by BioRed T20 cell counting system. Each experiment was independently repeated three times, each time in triplicate. *p < 0.05 *vs* HKC-2.

To examine the potential impacts of CD4+ T cells in the RCC development, we first tested whether RCC cells, compared to non-malignant kidney cells, could better attract the CD4+ T cells, HH, which were used in this study. We applied the *in vitro* transwell migration assay to study the effects of RCC cells on T cell recruitment. The T cells were then seeded in the upper transwells (pore size, 5 μm) and 2 different RCC cells 786-O and A498, or nonmalignant kidney HKC-2 cells were seeded in the bottom wells. After 6 hours of incubation, T cells that were attracted by RCC or non-malignant kidney cells and migrated into the bottom well were counted. The result (Fig. 1) revealed that RCC 786-O cells could recruit more T cells (2.1 ± 0.23 fold) than HKC-2. Compared to HKC-2 cells, A498 cells could better attract the T cells (∼1.7 ± 0.1 fold) (Fig. 1). All results have been repeated independently 3 times. Together, our data suggested that RCC cells could better attract CD4+ T cells than the non-malignant kidney cells.

### Recruited T cells enhanced the RCC cell invasion *via* up-regulation of ERβ signaling in RCC cells

**Figure 2:**
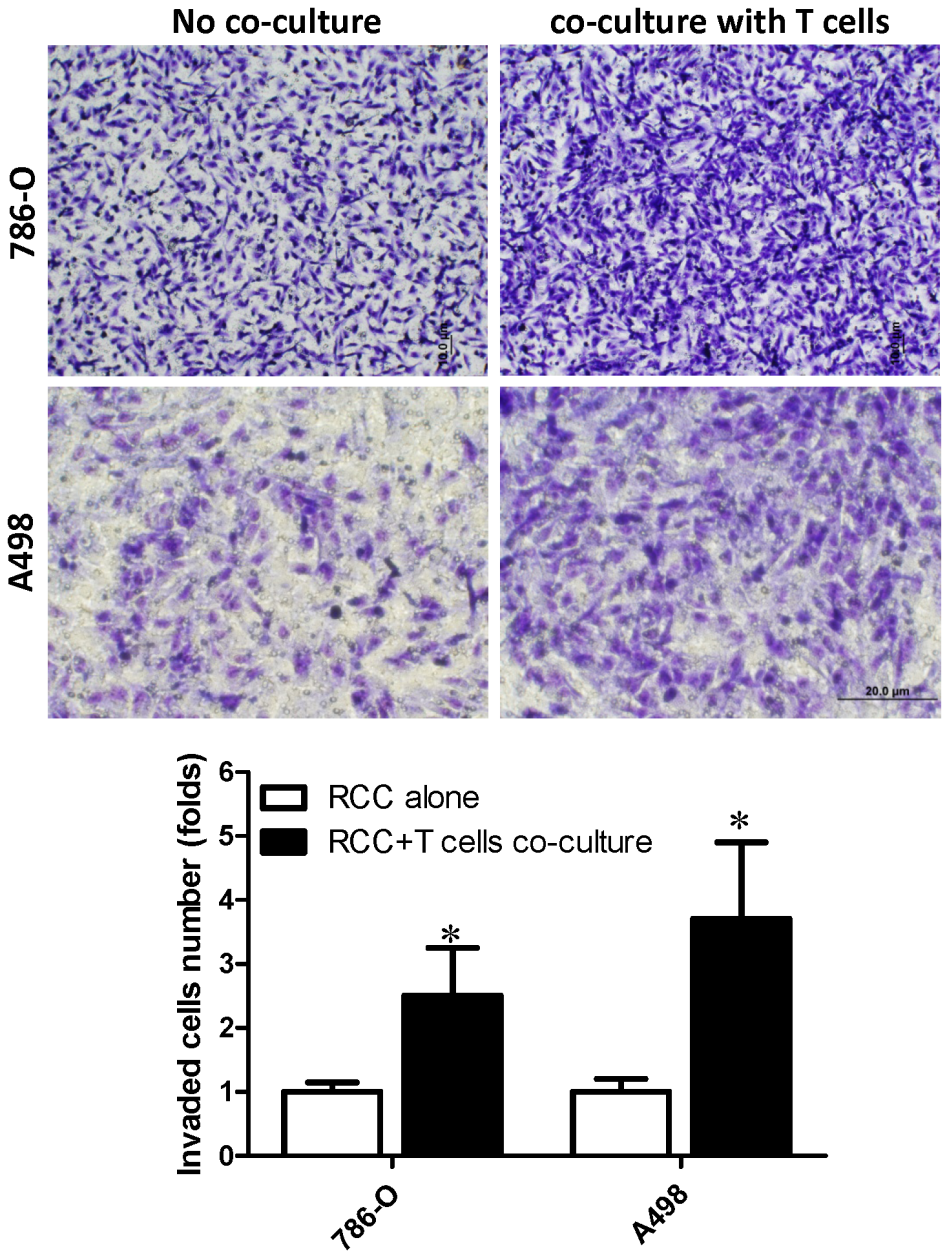
Recruited T cells could promote RCC cells invasion RCC cells, 786-O and A498, were cultured alone or co-cultured with T cells for 48 hrs. In a transwell system, the RCC cells with/without being co-cultured were re-seeded into the inserted wells that were pre-coated with matrigel for invasion assay. Invaded cells were stained by 1% toluidine blue (upper panels) and quantifications of invaded cells are presented in bottom panel. Each experiment was independently repeated three times, each time in triplicate. *p < 0.05 *vs* RCC alone.

To further study the consequences of recruited CD4+ T cells on RCC progression, we then applied the matrigel transwell invasion assay to test the invasion capability of RCC cells co-cultured with or without differentiated T cells for 2 days. The cells were then re-seeded in the upper transwell (5 × 10^4^/well). The invasion results showed that an increased invasion ability in RCC cells that have been co-cultured with T cells as compared with RCC without co-culture (Fig. 2). Co-culturing with T cells can increase 786-O cell invasion capability to 2.5 ± 0.75 fold and A498 cells to 3.7 ± 1.2 fold.

To dissect the potential mechanisms why recruited T cells can enhance RCC cell invasion, we examined several potential factors that could influence the RCC invasion. Those signal pathways include ERβ, VEGFA and HIF2α [20–22]. After characterization, we identified ERβ can be specifically up-regulated and the disabled homolog 2-interactiong protein (DAB2IP) can be specifically down-regulated in RCC cells after co-culture with T cells (refer to Fig. 4A). The pathways are specific as VEGFa and HIF2α did not change in RCC cells after co-culturing with T cells for 48 hrs (refer to Fig. 4A).

**Figure 3:**
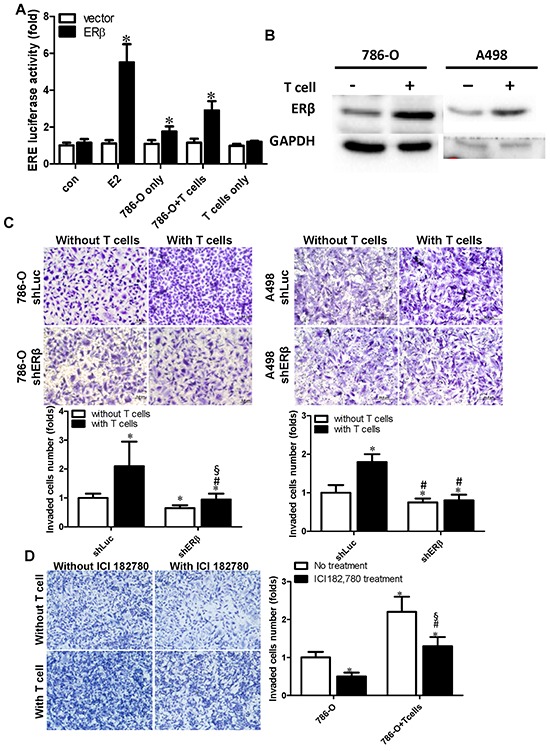
Co-culture of RCC and CD4+ T cells (HH) can activate ERβ transcriptional activity and increase ERβ expression in RCC cells **A.** ERβ transcriptional activity was increased by the conditioned media (CM) collected from co-cultured cells. 293T cells were transfected with ERβ, and (ERE)_3_-luciferase reporter plasmid by lipofetamine 3000 following the manufacture's protocol. CM was collected from RCC alone, T cells alone, or RCC+T cells co-culture and was used to treat 239T cells after 48 hrs transfection with ERβ and (ERE)_3_-luciferase reporter or control plasmids. 10 nM E2 treatment was applied as positive control for induction of ERβ activity. *p < 0.05 *vs* vector. **B.** Co-culture with T cells could increase ERβ protein expression in RCC cells. ERβ protein was detected by anti-ERβ polyclonal antibody (GTX 110607, GeneTex) at 1 to 1000 dilution in 1 × PBS with 5% BSA. *, #, § *p* < 0.05; * *vs* shLuc without T cell co-culture; # *vs* shLuc with T cells; § *vs* shERβ without T cell co-culture. **C.** T cells co-culture-induced ERβ can enhance RCC cell invasion. We introduced shERβ or shLuc in RCC cells, and then RCC cells were co-cultured with T cells. After incubating 48 hrs, RCC cells were re-seeded into upper wells of a transwell system to perform the matrigel invasion assay. * *vs* shLuc without T cell co-culture; # *vs* shLuc with T cell co-culture; *, # *p* < 0.05. **D.** Increased ERβ plays important roles in the T cells-enhanced RCC invasion. ER antagonist, ICI182,780 (1 μM), or mock control was used to treat 786-O cells for 24 hrs. Those 786-O cells were either cultured alone, or co-cultured with T cells for 48 hrs. RCC Cells were then seeded in the matrigel pre-coated insert wells for the transwell invasion assay. Each experiment was independently repeated three times, each time in triplicate. *, #, § *p* < 0.05 * *vs* 786-O no treatment; # *vs* 786-O+T cell no treatment T cell; § *vs* 786-O with ICI treatment

Among those changed factors, we focused on studying ERβ as recent reports indicated that ERβ could play important roles to influence the RCC cell invasion [20]. We first assayed the ERβ transactivation activity, Fig. 3A results revealed that E2 treatment, as a positive control, could activate ERβ transactivation in 293T cells by (ERE)_3_-Luciferase reporter assay. Furthermore, conditional conditioned media (CM) from co-cultured 786-O cells and T cells could better induce the (ERE)_3_-luciferase- activity by ∼2.9 fold compared to control media. The induction effect of CM from co-culture is also better than CM collected from 786-O cells only or T cells only (Fig. 3A)

In addition to observing that co-culture CM could stimulate the transactivation of ERβ, results from western blot analysis indicated that co-culturing RCC cells and T cells could increase ERβ protein expression in 786-O and A498 cells (Fig. 3B), suggesting that recruited T cells may promote RCC cell invasion *via* increasing the activity and expression level of ERβ. Importantly, using the interruption approach, with ERβ-shRNA to knock down ERβ mRNA, results revealed that knockdown of ERβ could block/reverse the recruited T cells-enhanced RCC cell invasion in both 786-O and A-498 cells (Fig. 3C). Similar results were also obtained when we replaced ERβ-shRNA with the anti-estrogen ICI182,780 showing the inhibition of ERβ activity by anti-estrogen could reverse recruited T cells-enhanced RCC cell invasion in 786-O (Fig. 3D).

Together, results from Fig. 3A–3D suggest that recruited T cells can enhance RCC cell invasion at least partly *via* increasing the ERβ expression and transactivation.

### Mechanisms study by which T cells can enhance ERβ expression and lead to the increased RCC cell invasion

**Figure 4:**
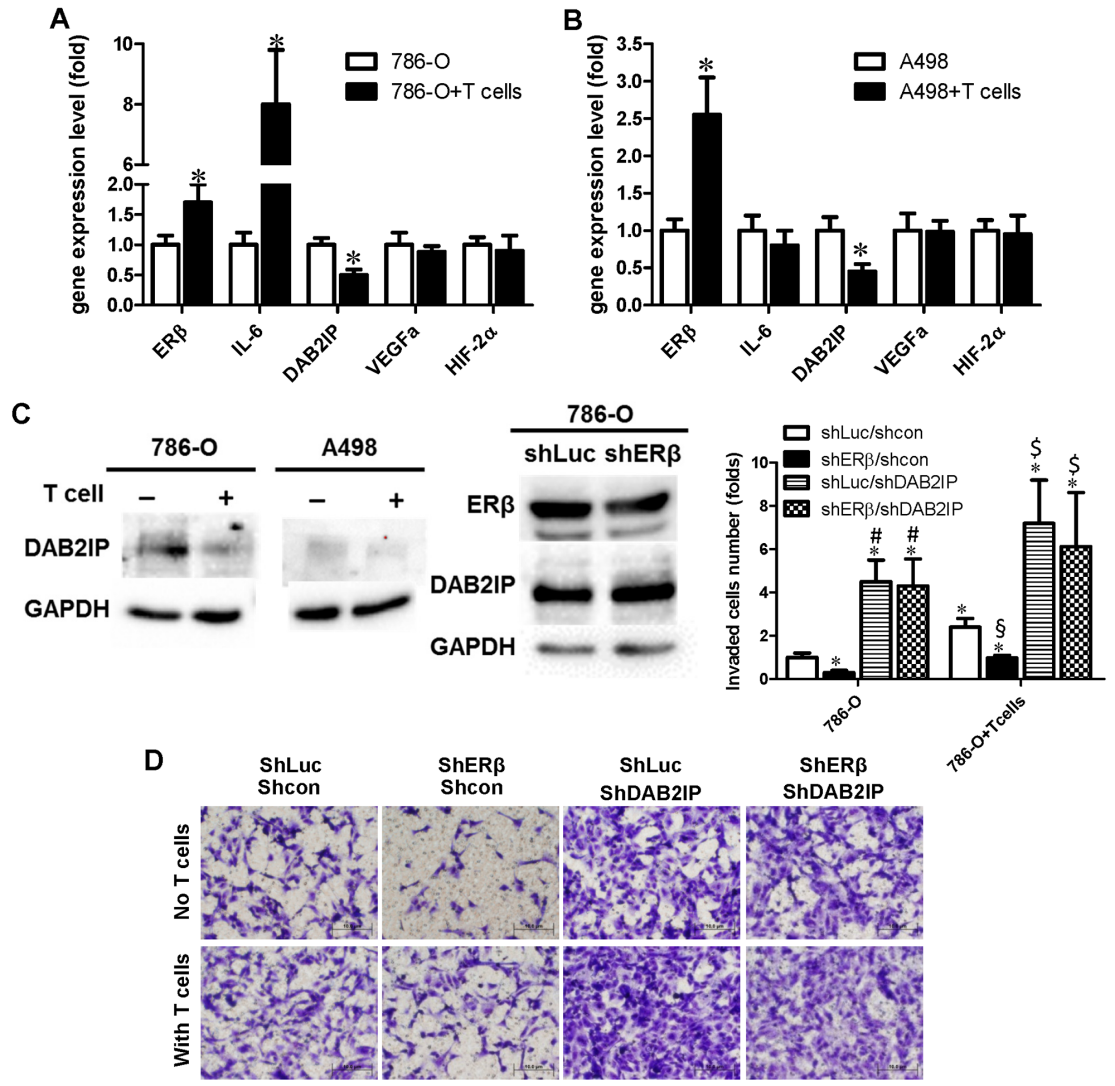
Recruited T cells can promote RCC cell invasion through ERβ/DAB2IP signal pathway **A–B.** Co-culture of RCC cells with T cells can increase ERβ, but decrease DAB2IP expression, in both 786-O and A498 cells. Total cell lysates were collected from RCC cells cultured alone or co-cultured with T cells for 48 hr. 50 μg of total protein was loaded in each well, anti-ERβ and anti-DAB2IP antibodies were used to detect their protein levels. *p < 0.05. **C.** ERβ could inhibit DAB2IP protein expression level in RCC. Total cell lysates were collected from RCC cells cultured alone or with T cells. DAB2IP expression was compared between these two conditions (left panels). ERβ could inhibit DAB2IP expression (right panels). DAB2IP protein level was compared between 786-O/sh control (shLuc) and 786-O/shERβ. **D.** ERβ-induced RCC invasion may function through down-regulation of DAB2IP. Lentiviral shERβ or shLuciferase (shLuc) was used to study the ERβ knockdown effects on RCC cell invasion. Our data showed that shERβ could reduce the RCC cell invasion. As ERβ knockdown can lead to the increased DAB2IP, we next tested whether knockdown of DAP2IP could reverse the ERβ knockdown-reduced RCC invasion. Lentiviral shDAB2IP or scramble control (shcon) were transduced into 786-O cells. 786-O cells express a high endogenous ERβ, thus 786-O cells are used to knock down ERβ for functional assay. After lentiviral transduction of different shRNA and controls, puromycin selection was used to enrich the transduced 786-O cells. Cells were then co-cultured with/without T cells, and then re-seeded to a new transwell matrigel invasion system to study the roles of ERβ and DAB2IP in 786-O cells invasion. The invaded 786-O cells were stained with 1% toluidine B in PBS (bottom panel). Quantifications of invaded 786-O cells were shown in right panel. *, #, § and $ *p* < 0.05 * *vs* shLuc/sh cp mom 786-O; # shERβ/shcon in 786-O; § *vs* shLuc/shcon in 786-O+T cells; $ *vs* shERβ/shcon in 786-O+T cells.

To further dissect the molecular mechanism(s) that are responsible for T cells-enhanced ERβ expression and increased RCC cell invasion, we applied Q-PCR-based focus-array to quantify the expressions of several key metastasis-related genes. Among those altered metastasis-related genes, we found the expression of DAB2IP was decreased when ERβ expression was increased in RCC 786-O and A498 cells (Fig. 4A and 4B). DAP2IP has been reported to function as a suppressor for cancer progression [23–25]. Consistent with mRNA level changes, using Western Blot analyses we also found co-culturing RCC cells with T cells could up-regulate ERβ in RCC cells (see Fig. 3B) and down-regulate DAB2IP (Fig. 4C, left panels). Importantly, we observed that knockdown of ERβ by shRNA up-regulated DAB2IP expression in RCC 786-O cells (Fig. 4C, right panels).

To further investigate the functions and the correlation of DAB2IP in ERβ-promoted RCC invasion, we knocked down DAB2IP in 786-O cells that have been transduced with lentiviral shERβ and the results showed that knockdown of DAB2IP can reverse shERβ-reduced 786-O invasion (Fig. 4D).

Data presented in Fig. 4 suggested that co-culturing T cells with RCC cells could increase ERβ signals in RCC and the increased ERβ negatively regulates the expression of tumor suppressor DAB2IP. Together, the recruited T cells can enhance RCC cell invasion *via* altering the ERβ → DAB2IP signals.

### Mechanisms by which tumor cells attract T cells into the RCC tumor microenvironment

**Figure 5:**
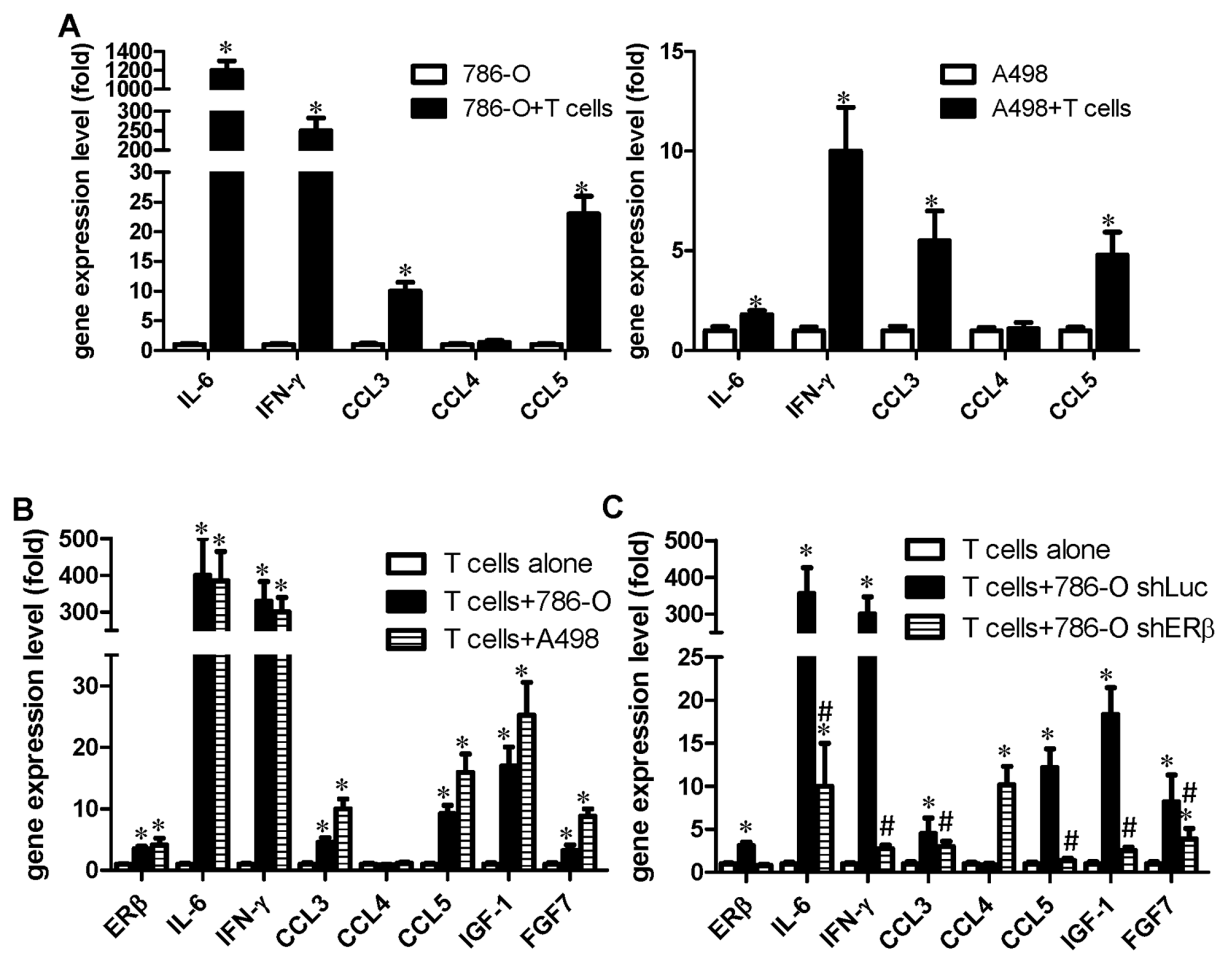
RCC cells promote T cells to express more IGF, FGF and IFN-γ, which could then activate the ERβ pathway in RCC cells RCC and T cells were co-cultured for 48 hrs, the RCC or T cells were separated, and then metastasis-related gene expressions were analyzed by Q-PCR. **A.** RCC cells (with T cells) vs RCC cells (only). *p < 0.05. **B.** T cells (with RCC co-culture) vs T cells (only). **C.** ERβ in RCC could affect T cells activity. RCC cells with/without ERβ knockdown cultured alone, or co-cultured with T cells. After 48 hrs incubation, target genes were analyzed in T cells. *, # *p* < 0.05 * *vs* T cell alone; # *vs* T cell+786-O shLuc

We further studied why RCC can recruit T cells into the tumor microenvironment. We hypothesize a positive regulatory feed-back mechanism might exist. We first analyzed T cells recruitment related cytokines [26] in RCC cells after co-culture with T cells. The results indicated IFN-γ, CCL3 and CCL5 were increased in both RCC cells after co-culture (Fig. 5A). Interestingly and importantly, the expressions of IFN-γ, CCL3 and CCL5 were also increased in T cells after co-cultured with 786-O or A498 RCC cell lines (Fig. 5B). In addition, we found knockdown of ERβ in 786-O cells, that have been pre-incubated with T cells for 48 hrs, could suppress the infiltrating T cells mediated increase of IFN-γ, CCL3 and CCL5 in 786-O cells (Fig. 5C).

Together, these results indicate that the recruited T cells could function *via* increasing IFN-γ expression in both RCC and T cells using a positive regulatory feed-back mechanism to enhance T cells migration into the RCC tumor microenvironment. Importantly, the recruited T cells could subsequently increase ERβ expression in RCC cells to promote RCC cell invasion, and the increased ERβ in RCC cells could further increase IFN-γ expression in T cells to further promote T cells recruitment, by a positive-feedback mechanism.

In addition to IFN-γ, we found that the co-culture of T cells with RCC cells could increase the expression of IGF-1 and FGF7 in T cells (Fig. 5B), and knocking down ERβ in 786-O cells could reverse the T cells/RCC cells co-culture-enhanced IGF-1 and FGF-7 production from T cells (Fig. 5C). This is important since growth factor signals could activate ERs transcriptional activity [27]. It will be interesting to test if T cells may also function through secretion of IGF-1 and FGF7 to increase RCC ERβ in a positive feedback mechanism to enhance RCC cell invasion.

**Figure 6:**
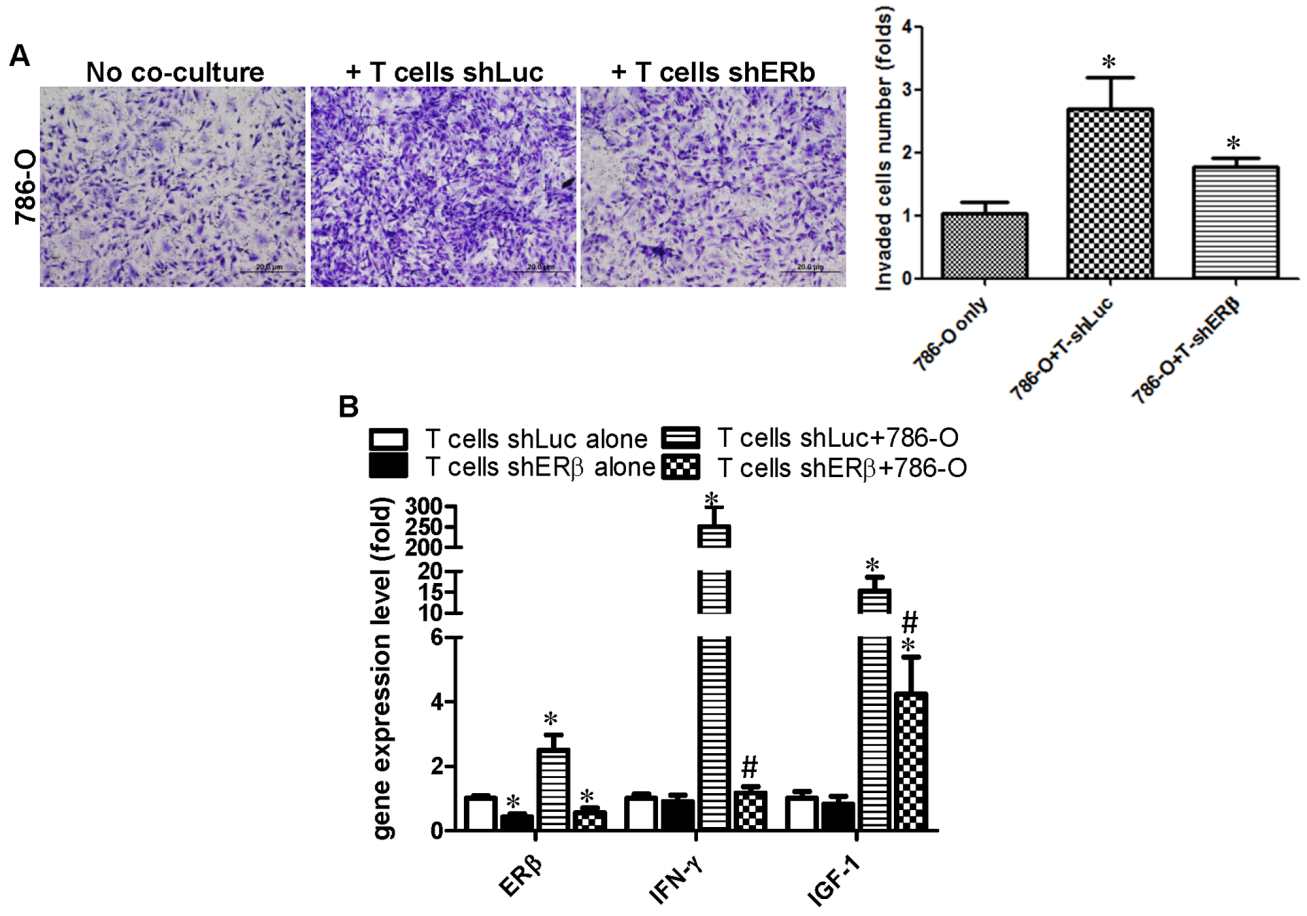
Co-culture of RCC cells with T cells can enhance IFN-γ and IGF production in T cells *via* increasing ERβ in T cells T cells transduced with lentiviral ERβ or shLuc were subjected to puromycin selection to establish the stable clones in T cells. **A.** T cells, with shERβ or with shLuc, were co-cultured with 786-O cells to test the invasion capability. * *P* < 0.05, *vs 786-O cells only. **B.** ERβ, IFN-γ and IGF-1 gene expression in T cells (shLuc, shERβ) alone and co-cultured with RCC cells. Each experiment was independently repeated 3 times. *, # *p* < 0.05, *vs T cells shLuc alone; # *vs* T cell shLuc+786-O; *p* ≤ 0.05 was considered statistically significant.

Furthermore, we obtained similar results in T cells, knocking down ERβ in T cells could consequently suppress the co-cultured RCC cells invasion (Fig. 6A), and knocking down ERβ in T cells could also reverse the T cells/RCC co-culture-enhanced IFN-γ and IGF-1 in T cells (Fig. 6B).

## DISCUSSION

RCC is the major type (85–90%) of renal cancer and is categorized as a highly vascularized tumor with high metastatic incidence. Based on the clinical cases analysis, there are 25%–33% of RCC patients who develop metastatic disease, and 20%–40% of patients who undergo treatment for localized disease will eventually develop metastases [28]. More than 50% of patients with early stage RCC are cured by surgery to remove the tumor and the 5-year survival rate is around 74–81%. However, the outcome for that late stage RCC is only around 8%, based on the updated website information of the American Cancer Society. It is important to seek new therapeutic strategies to treat RCC patients. Our study indicates T cells can promote RCC invasion *via* increasing the expression of ERβ and decreasing DAB2IP in RCC cells.

In addition to tumor cells, the tumor tissues also include immune cells and several types of secretion factors, including growth factors, cytokines, chemokines, and components of the extracellular matrix. The interaction between immune cells and cancer malignant cells is a complicated process and the immune system may either promote or inhibit cancer progression [29]. A recent study indicated that infiltrated immune cells can promote cancer progression and invasion [30]. A growing body of evidence showed that the existence of regulatory T cells (Treg) may induce tumor cells to change cell secretary profiles and to develop abilities to successfully evade the host immune system [31–33]. Meanwhile, the increased numbers of recruited T cells and mast cells in tumor tissues are also correlated with tumor progression and have been demonstrated to be prognostic factor(s) in predicting clinical outcomes [34, 35]. In RCC, Polimeno *et al*. indicated that Treg cells CD4+/CD25(high+)/FOXP3+ (forkhead box protein 3) were greater in the patients with RCC (*p* < 0.001) than other types of immune cell cancers. Meanwhile, Kaplan-Meier analysis for disease-free survival showed that high numbers of Treg cells predict short disease-free survival in patients with RCC [36]. This result indicates the important roles of Tregs in the suppression of the anti-tumor immune response. It is well known that CD4+CD25+ and FOXP3 are major markers for Treg cells. Although HH cells have been identified as CD4+ but CD25-, inducible Tregs (iTregs) originate as CD4+ cells from the thymus, and following adequate antigenic stimulation in the presence of cognate antigen and specialized immunoregulatory cytokines, such as TGF-β, IL-10, and IL-4; Tregs can be differentiated into CD25+ and FOXP3+ expressing Tregs hence are called “adaptive” or “inducible” Tregs [37]. The HH cells we used could be transformed to Treg cells and promote RCC progression. Indeed, our unpublished data showed that the CD25 expression was increased in HH cells after co-culture with RCC cells, indicating that the interaction between RCC cells and T cells may facilitate the transformation of HH cells to Treg-like cells.

DAB2IP is a member of the RasGTPase-activating protein family. It interacts directly with DAB2, which suppresses the growth of many cancer types [38, 39]. DAB2IP can suppress the PI3K/Akt pathway and enhance ASK1 activation leading to cell apoptosis. In metastatic prostate cancer (PCa), DAB2IP expression is often down-regulated and results in PI3K/Akt activation and ASK1-JNK inactivation leading to accelerated PCa growth [40]. In addition to its role in PCa, down-regulation of DAB2IP increased cell proliferation and invasion in bladder cancer [41]. A recent study also showed the loss of DAB2IP gene expression in different subtypes of renal cell carcinoma [42]. Hsieh's study indicated that DAB2IP was detected in 95% (38/40) of normal kidney tissues, but decreased or loss of DAB2IP expression was detected in 56.6% (159/281) of RCC tissues. In knocked down DAB2IP RCC cell lines, HIF-α expression, important in promoting RCC metastasis, was increased. DAB2IP, as a novel tumor suppressor, could prevent RCC metastasis *via* inhibiting HIF-α [42]. To the best of our knowledge, our study is the first to demonstrate the correlation between ERβ and DAB2IP. DAB2IP expression was down-regulated when T cells increased the ERβ expression in RCC cell lines. Our data demonstrate that the increased ERβ expression could diminished DAB2IP protein levels. However, there was no evidence that ERβ binds directly to DAB2IP. Previous studies have demonstrated EZH2 can decrease DAB2IP expression [43] and E2 can increase EZH2 through ERs and tumor progression [44]. Together, these results demonstrate E2 may promote cancer progression through the ERs/EZH2/DAB2IP pathway.

Our data showed that IFN-γ, CCL3 and CCL5 expression levels increased in both T cells and RCC cells after co-culture. A previous study demonstrated that increasing IFN-γ can enhance T cells migration to tumors [45]. Meanwhile, *in vivo* animal studies showed that co-injection of IFN-γ with other inflammatory factors can increase T lymphocyte migration *via* up-regulating the expression of CXCL10 or CCL5 [26]. There are two types of chemokine receptors, CCR5 and CXCR3, involved in T cell recruitment [38, 46–48] and it is well-known that IFN-γ can increase CCR5 expression in several types of cells [49], including CD4+ T cells [50]. The CCR5 ligands, including CCL3, CCL4 and CCL5, can conjugate with CCR5 and may enhance the attraction of T cells. CCL3 is also involved in CD4+ T cell recruitment [51] and is very efficient in recruiting T cells [52]. In sarcoidosis patients, sarcoidosis increases the attraction of CD4+ T cells through the release of CCL4, either alone or together with Th1/Tc1-associated cytokines [53]. However, our results showed that co-culturing RCC with T cells only increased CCL3 and CCL5, but not CCL4. Interestingly, although CCL4 expression did not change in RCC cells or T cells after co-culture, knocking down ERβ in 786-O cells and then co-culturing with T cells can lead to an increased CCL4 expression in the T cells. It has been known that the miR-125b can down-regulate CCL4 expression in monocytes and T cells by binding of the 3′UTR of CCL4 [54]. Meanwhile, activating transcription factor 3 (ATF3) also was found to repress the expression of CCL4 in murine macrophages [55]. Both studies showed the possible mechanisms involved in the down-regulation of CCL4 in T cells. According to our results, ERβ in RCC cells may function through paracrine factors to increase miR-125b, and subsequently decrease CCL4 in T cells. Therefore, we could observe the increased CCL4 expression in T cells when ERβ was knocked down in RCC cells. This may require more detailed experimental study in the future regarding the mechanisms and clinical significance about this ERβ-regulated CCL4 in the RCC tumor microenvironment.

**Figure 7:**
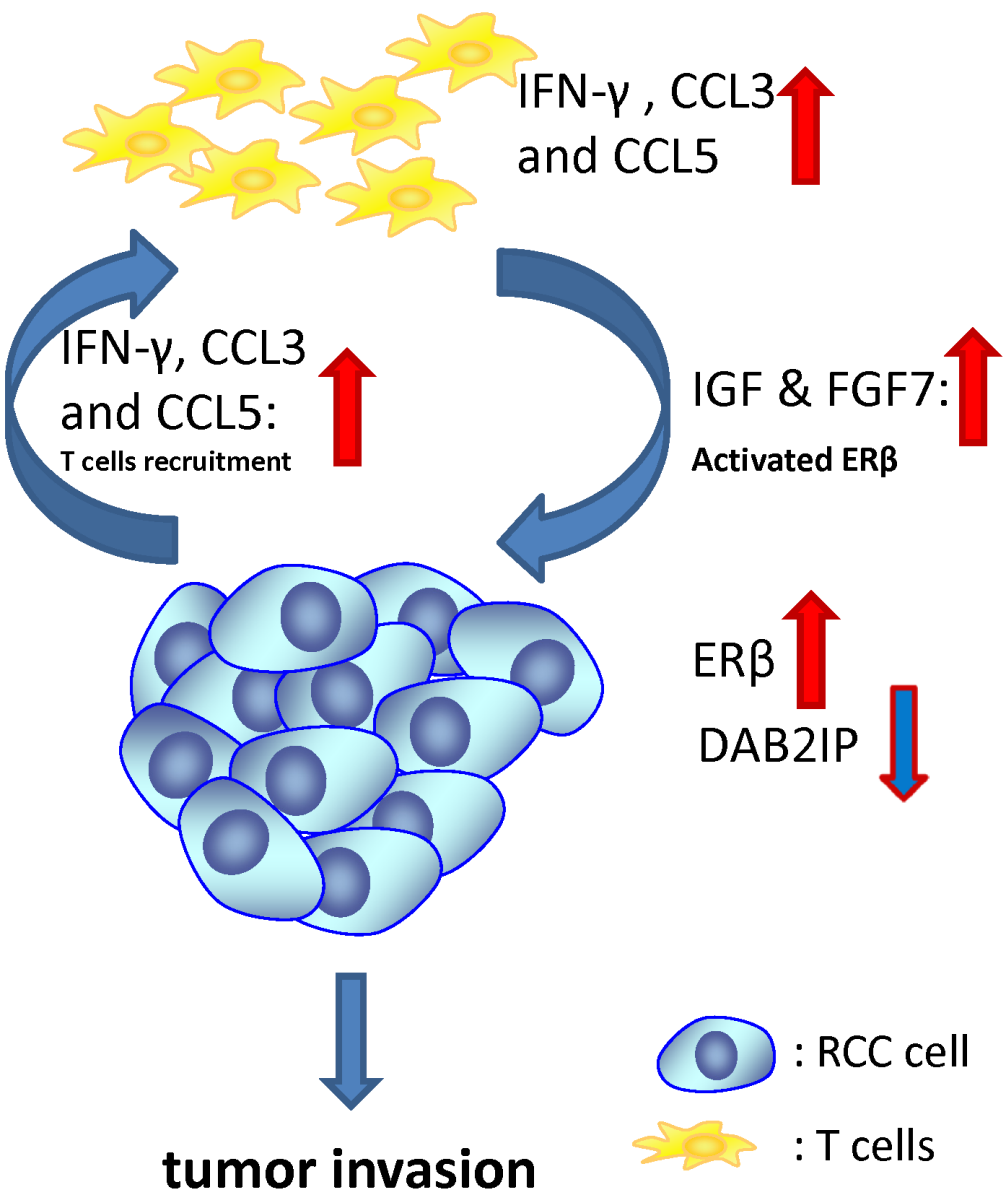
Schematic presentation of the interaction mechanisms of T cells with RCC cells in the tumor microenvironment After T cells migrated toward RCC cells, both ERβ transcriptional activity and protein expression increase, but DAB2IP expression decreases in RCC cells. In addition to ERβ, IFN-γ, CCL3, CCL5, IGF and FGF7 also increased in both RCC and T cells after co-culture. IFN-γ and CCL3 and CCL5 may correlate with T cell recruitment. In addition to E2, IGF and FGF7 have been demonstrated to activate ERs transactivation. All these increased genes were regulated by ERβ when the infiltrated T cells interact with RCC cells in the tumor microenvironment.

We also demonstrated the CM from RCC alone can slightly activate ERβ transcriptional activity and CM from RCC and T cells can further enhance this effect (Fig. 3). After studying the gene profiles, we found IGF expression increased in both RCC and T cells after co-culture and knock down of ERβ can reverse co-cultured induced IGF expression (Figs. 6 and 7). A previous study indicated that IGF-1 can activate EREs through ERs mediated transcriptional activation in breast cancer [27]. Meanwhile, IGF also can conjugate with IGF-1R (IGF-1 receptor) and insulin receptors to modulate ERα and ERβ translocation to the nuclei, membrane organelles, and the mitochondria *via* an estrogen independent pathway [56]. These results indicated that recruited T cells have the potential to activate ERβ in RCC cells and promote RCC progression.

We demonstrated RCC cells have better T cell recruitment ability compared to normal kidney cells and the consequence of T cell recruitment could enhance RCC invasion (Fig. 7). Mechanistic studies showed that after T cells migrated toward RCC cells, both ERβ transcriptional activity and protein expression increased, but DAB2IP, a tumor suppressor gene, decreased. In addition to ERβ, IFN-γ, CCL3, CCL5 and IGF also increased in both RCC and T cells after co-culture. IFVγ, CCL3 and CCL3 and CCL5 may correlate with T cell recruitment and IGF, instead of E2, has been demonstrated able to activate ERs activity. All these increased genes were regulated by ERβ. The newly identified relationship between T cells and RCC cell ERβ/DAB2IP pathway may help to establish a new biomarker for predicting the RCC progression, as well as a therapeutic target in the development of agents against RCC.

## MATERIALS AND METHODS

### Cell lines

Two RCC cell lines, 786-O and A498, and CD4+ T cells were purchased from the American Type Culture Collection (ATCC) (Rockville, MD). 786-O and A498 cells were maintained in DMEM media with 10% fetal bovine serum (FBS) and 1% penicillin/streptomycin. CD4+ T cell was maintained in DMEM with 10% heat inactivated FBS with 1% penicillin/streptomycin. CD4+ T cells were differentiated by treating with 1% dimethylsulfoxide (DMSO) for one week before used in experiments. DMSO or phorbol esters is often used to differentiate immune cells [57, 58].

### Cell migration assay

786-O and A498 and normal human kidney proximal tubular cell line, HKC-2, at 1 × 10^5^, were plated into the lower chambers of the transwells with 5 μm pore polycarbonate membrane inserts. 1 × 10^5^ differentiated CD4+ T cells (HH) were plated onto the upper chambers. After 6 hrs, the cells migrated into the lower chambers were collected and counted by the Bio-Rad TC10 automatic cell counter. Each data point was performed in triplicate and the experiments were independently repeated twice.

### Invasion assay

The effects of T cells in RCC cell invasion capability was determined using the transwell invasion assay. 786-O and A498 cells were co-cultured with CD4+ T cell for 48 hrs. To set up the cell co-culture, T cells were seeded into the upper chamber (pore size 0.4 μm) at 1.5 × 10^4^ cells/chamber and co-cultured with RCC cells at 1.5 × 10^5^/well with DMEM including 10% heat inactivated FBS in lower Chamber. After co-cultured for 48 hr, RCC cells were trypsinized and reseeded into transwells that were pre-coated with matrigel (0.2 mg/ml; 100 μl/well) for 24 hrs to determine their invasion ability. The invaded RCC cells attached to the lower surface of the membrane were fixed by 75% ethanol and stained with 1% toluidine blue. Cell numbers were counted in five randomly chosen microscopic fields per membrane.

### Estrogen response element luciferase promoter assay

(ERE)_3_ luciferase activity was performed using Lipofectamine 3000 (Invitrogen). 293T cells were transfected with 0.1 μg pcDNA ERβ, 0.4 μg (ERE)_3_-Luc and 1 ng pRL-TK-Luc reporter gene. After transfection, the media were refreshed to 10% charcoal/dextran stripped-FBS media for 24 hrs and CM collected from RCC and T cells co-cultured system was added as indicated for another 24 hrs. Cells were then harvested for the dual luciferase assay (Promega, Madison, WI).

### Plasmids and ientivirus infection

To overexpress ERβ, cDNA of ERβ was cloned into pWPI vector and to silence ERβ expression, the shERβ sequence was cloned into PLKO.1 backbone. The 293T packaging cells were transiently transfected with pMD2.G and psPAX2 with PLKO.1-shluc/shERβ or PLKO.1-control/shDAB2IP (kindly provided by Prof. Jer-Tsong Hsieh, UT Southwestern Medical Center at Dallas), to produce lentiviral particles. The supernatants containing lentiviral particles were collected 48 hrs post-transfection of 293T cells, filtered and used to transduce RCC cells for 48 hrs with polybrene. The viral transduced RCC cells were then subjected to 1 μg/ml puromycin selection. The detailed procedure was described in our previous published paper [59].

### RNA extraction and quantitative real-time PCR analysis

Total RNA was extracted by Trizol reagent (Invitrogen, CA) according to the manufacturer's instructions. RNAs (1 μg) were subjected to reverse transcription using Superscript III transcriptase (Invitrogen). The obtained cDNAs were applied for qPCR using a SYBR green Bio-Rad CFX96 system. The primers used are in Table 1. RNA expression levels were normalized to the expression of GAPDH.

**Table 1:** Primer sequences for quantitative PCR

Gene	Accession No.	sequences
GAPDH	NM_002046	Forward: 5′-GGAGCGAGATCCCTCCAAAAT-3′ Reverse: 5′-GGCTGTTGTCATACTTCTCATGG-3′
ERβ	NM_001214902	Forward: 5′-TCCATCGCCAGTTATCACATCT-3′ Reverse: 5′-CTGGACCAGTAACAGGGCTG-3′
DAB2IP	NM_138709	Forward: 5′-CTGAGCGGGATAAGTGGATGG-3′ Reverse: 5′-AAACATTGTCCGTCTTGAGCTT-3′
HIF-2α	NM_001001392	Forward: 5′-GTGCTCCCACGGCCTGTA-3′ Reverse: 5′-TTGTCACACCTATGGCATATCACA-3′
VEGFa	NM_001171627	Forward: 5′-AGGGCAGAATCATCACGAAGT-3′ Reverse: 5′-AGGGTCTCGATTGGATGGCA-3′
IL-6	NM_000600	Forward: 5′-CCTGAACCTTCCAAAGATGGC-3′ Reverse: 5′-TTCACCAGGCAAGTCTCCTCA-3′
IFN-γ	NM_000619	Forward: 5′-TCGGTAACTGACTTGAATGTCCA-3′ Reverse: 5′-TCGCTTCCCTGTTTTAGCTGC-3′
CCL3	NM_002983	Forward: 5′-AGTTCTCTGCATCACTTGCTG-3′ Reverse: 5′-CGGCTTCGCTTGGTTAGGAA-3′
CCL4	NM_002984	Forward: 5′-CTGTGCTGATCCCAGTGAATC-3′ Reverse: 5′-TCAGTTCAGTTCCAGGTCATACA-3′
CCL5	NM_002985	Forward: 5′-CCAGCAGTCGTCTTTGTCAC-3′ Reverse: 5′-CTCTGGGTTGGCACACACTT-3′
IGF-1	NM_001111283	Forward: 5′-GCTCTTCAGTTCGTGTGTGGA-3′ Reverse: 5′-GCCTCCTTAGATCACAGCTCC-3′
FGF-7	NM_002009	Forward: 5′-TCCTGCCAACTTTGCTCTACA-3′ Reverse: 5′-CAGGGCTGGAACAGTTCACAT-3′

### Western blot assay

Total protein was extracted by RIPA buffer containing 1% protease inhibitors (Amresco, Cochran, USA). The following primary antibodies were used: rabbit anti-ERβ polyclonal antibody (GTX 110607, GeneTex), mouse anti-DAB2IP polyclonal antibody (1 μg/ml) and and mouse anti-GAPDH monoclonal antibody (Santa Cruz), all used at 1:1000 dilution. The immune-positive bands were visualized with an ECL chemiluminescent detection system (Thermo Scientific) and the images were transferred to the Bio-Rad imaging system. All analyses were performed at least in duplicate. The detailed procedure of Western blot was described in our previous publication [60].

### Statistical analysis

Values were expressed as mean ± standard deviation (SD). The Student's t test and one-way NOVA were used to calculate two-sided *P* values, and considered statistically significant when *P* < 0.05.
